# Correction: Development and psychometric properties of Iranian midwives job satisfaction instrument (MJSI): A sequential exploratory study

**DOI:** 10.1371/journal.pone.0316910

**Published:** 2024-12-31

**Authors:** Ashraf Direkvand-Moghadam, Nasrin Rashan, Mona Bahmani, Safoura Taheri

There are errors in the author affiliations. The correct affiliations are as follows:

Ashraf Direkvand-Moghadam^1^, Nasrin Rashan^2^, Mona Bahmani^2^, Safoura Taheri^1^

**1** Department of Midwifery, Faculty of Nursing and Midwifery, Ilam University of Medical Sciences, Ilam, Iran, **2** Student Research Committee, Ilam University of Medical Sciences, Ilam, Iran.
