# Supplementary material for: Development and psychometric properties of Iranian midwives job satisfaction instrument (MJSI): A sequential exploratory study
Source: PLoS One. 2022 Jan 25;17(1):e0262665. doi: 10.1371/journal.pone.0262665 (PMC8789179; doi:10.1371/journal.pone.0262665)
Supplement: S1 File — (DOCX) [file pone.0262665.s001.docx]

**Questions guide in Qualitative phase**

In Qualitative phase, the interview guide included the following questions that were identified as the progress of the study and data collection, the simultaneous analysis and the creation of subcategories of the next interview route.

1. Are you satisfied with your job?
2. What do you think job satisfaction means? What is your perception of job satisfaction?
3. What comes to mind when you hear the word job satisfaction?
4. What can affect your job satisfaction or dissatisfaction?
5. What are your criteria for job satisfaction and dissatisfaction?
6. Please explain to me? Please tell me about your experience?
7. Probing Questions: Can you explain more about this? Can you give an example? "What do you mean?".
