# Supplementary material for: Development and psychometric properties of Iranian midwives job satisfaction instrument (MJSI): A sequential exploratory study
Source: PLoS One. 2022 Jan 25;17(1):e0262665. doi: 10.1371/journal.pone.0262665 (PMC8789179; doi:10.1371/journal.pone.0262665)
Supplement: S4 File — (DOCX) [file pone.0262665.s004.docx]

**The Iranian midwives job satisfaction instrument(MJSI)**

**The job satisfaction questionnaire**

While wishing you a good health, we would like to inform you that the questionnaire in front of you is used to collect information about “job satisfaction of midwives working in the city of Ilam in 2019". The questionnaire will take approximately 10 minutes to complete. Your accuracy in answering will help us to make proper analysis and provide logical solutions. It should be noted that, the information contained in the questionnaire is completely confidential and its results are only used to analyze the current situation. Thank you in advance for your cooperation.

| Factors name |  | **Items** | **Very little or nothing** | **Little** | **Somewhat** | **Very** | **Too much** |
| --- | --- | --- | --- | --- | --- | --- | --- |
| Communications dimension |  | How is your relationship with the midwifery team (faculty, trainers, and faculty members)? |  |  |  |  |  |
|  |  | How is your relationship with midwife colleagues at other health care centers, organizations, etc.? |  |  |  |  |  |
|  |  | How is your relationship with midwives working in the NGO sector? |  |  |  |  |  |
|  |  | How is your relationship with GPs? |  |  |  |  |  |
|  |  | How is your relationship with gynecologists? |  |  |  |  |  |
|  |  | How is your relationship with pediatricians? |  |  |  |  |  |
|  |  | How do you relate to those around you and ordinary people? |  |  |  |  |  |
| Professional dimension |  | Does midwifery meet the economic needs of a middle-aged person? |  |  |  |  |  |
|  |  | How is job security in the midwifery profession in terms of the labor market? |  |  |  |  |  |
|  |  | What is midwifery occupational safety? (Protecting employees from wage and salary fluctuations and eventually losing their jobs) |  |  |  |  |  |
|  |  | What is the flexibility of a midwife's work hours? |  |  |  |  |  |
|  |  | Do you think it is possible to improve practical skills in the midwifery profession? |  |  |  |  |  |
|  |  | How much can you use your knowledge and skills in service delivery? |  |  |  |  |  |
|  |  | In your opinion, how much is your workload compared to the working time during each shift? |  |  |  |  |  |
|  |  | How useful is the midwifery profession's usefulness in a midwife's life? |  |  |  |  |  |
|  |  | How much time can you upgrade your professional knowledge and skills? |  |  |  |  |  |
|  |  | How much can you upgrade your professional knowledge and skills in terms of available resources? |  |  |  |  |  |
| Responsibility dimension |  | How are you responsible in the midwifery profession? |  |  |  |  |  |
|  |  | How much do you have responsibility for your career after the end of your shift? |  |  |  |  |  |
| Physical-Mental dimensions |  | How much do you want to stay in the midwifery profession in the future? |  |  |  |  |  |
|  |  | How much the midwifery profession affect midwife's mental health? |  |  |  |  |  |
|  |  | How much is leisure time in the midwifery profession? |  |  |  |  |  |
|  |  | How much the midwifery profession affect midwife's physical health? |  |  |  |  |  |
| Social dimension |  | How do you evaluate the social status of the midwifery profession in the community? |  |  |  |  |  |
|  |  | How do you evaluate the social acceptance of the midwifery profession in the community? |  |  |  |  |  |

**©** [Direkvand-Moghadam](https://www.ncbi.nlm.nih.gov/pubmed/?term=Direkvand-Moghadam%20A%5BAuthor%5D&cauthor=true&cauthor_uid=22717414) A. et al., 2019
