# Supplementary material for: Development and psychometric properties of Iranian midwives job satisfaction instrument (MJSI): A sequential exploratory study
Source: PLoS One. 2022 Jan 25;17(1):e0262665. doi: 10.1371/journal.pone.0262665 (PMC8789179; doi:10.1371/journal.pone.0262665)
Supplement: S5 File — (DOC) [file pone.0262665.s005.doc]

**پرسشنامه رضایت شغلی ماماهای ایرانی**

ضمن آرزوی سلامتی برای شما، به اطلاع می رساند پرسشنامه ای که پیش روی شماست، به منظور جمع آوری اطلاعات در خصوص"بررسی رضایت شغلی ماماهای شاغل در شهر ایلام در سال 1398" می باشد. تکمیل پرسشنامه تقریبا 10 دقیقه طول خواهد کشید. دقت شما در پاسخگویی، ما را در تحلیل و نتیجه گیری مناسب و ارائه راه حل های منطقی یاری خواهد کرد. لازم به ذکراست که اطلاعات مندرج در پرسشنامه کاملا محرمانه بوده و از نتایج آن به صورت کلی و صرفاً برای تجزیه و تحلیل وضعیت موجود استفاده می شود. پیشاپیش از همکاری شما کمال تشکر و قدردانی را دارم.

| خیلی زیاد | زیاد | تا حدودی | کم | خیلی کم یا هیچ | گویه | فاکتور | ردیف |
| --- | --- | --- | --- | --- | --- | --- | --- |
|  |  |  |  |  | 1.ارتباط شما با همکاران گروه مامایی( اساتید، مربیان و همکاران دانشکده) چگونه است؟ | ارتباطی | 1 |
|  |  |  |  |  | 2.ارتباط شما با همکاران مامایی سایر مراکز بهداشتی، درمانی، سازمان ها و ... چگونه است؟ |
|  |  |  |  |  | 3.ارتباط شما با دیگر ماماهای شاغل در سازمان های غیر دولتی چگونه است؟ |
|  |  |  |  |  | 4.ارتباط شما با پزشکان عمومی چگونه است؟ |
|  |  |  |  |  | 5. ارتباط شما با متخصصان زنان و زایمان چگونه است؟ |
|  |  |  |  |  | 6. ارتباط شما با متخصصان اطفال چگونه است؟ |
|  |  |  |  |  | 7. ارتباط شما با بازرسان حوزه کاری چگونه است؟ |
|  |  |  |  |  | 8.چقدر از مهارت هایی حرفه ای تان استفاده میکنید؟ | ماهییت حرفه ای | 2 |
|  |  |  |  |  | 9.اشتغال در حرفه مامایی، نیازهای اقتصادی یک زندگی متوسط را تامین می کند؟ |
|  |  |  |  |  | 10.از نظر شما، امکان ارتقای مهارت های عملی در حرفه مامایی وجود دارد؟ |
|  |  |  |  |  | 11.چقدر می توانید از دانش و مهارت هایتان در ارائه خدمات استفاده کنید؟ |
|  |  |  |  |  | 12.از نظر منابع در دسترس، چقدر می توانید دانش و مهارت های حرفه ای تان را ارتقاء ببخشید؟ |
|  |  |  |  |  | 13.کاربرد مفید حرفه ی مامایی در زندگی یک ماما چه قدر است؟ |
|  |  |  |  |  | 14.از نظر شما، در هر شیفت کاری، حجم کارتان در مقایسه با مدت زمان کار چگونه است؟ |  |  |
|  |  |  |  |  | 15.از نظر فرصت زمانی، چقدر می توانید دانش و مهارت های حرفه ای تان را ارتقاء ببخشید ؟ |
|  |  |  |  |  | 16.اوقات فراغت (فرصتی ست که انسان موظف به انجام هیچ گونه تکلیف یا کار خاصی نیست جز اموری که با میل و انگیزه شخصی باشد) در حرفه ی مامایی در چه حد است؟ |
|  |  |  |  |  | 17.انعطاف ساعت کاری حرفه مامایی(سیاست کاری ساعت منعطف به کارکنان اجازه می دهد که چه زمانی کار کرده و چه زمانی به کار خود خاتمه دهند؟) در چه حد است؟ |
|  |  |  |  |  | 18.مسئولیت پذیری شما در حرفه مامایی در چه حد است؟ | مسئولیت پذیری | 3 |
|  |  |  |  |  | 19.بعد از پایان شیفت کاری، احساس مسئولیت در مورد حرفه تان دارید؟ |
|  |  |  |  |  | 20.امنیت شغلی ( محافظت از کارمندان در برابر نوسانات حقوق و درآمد و در نهایت از دست دادن جایگاه) درحرفه ی مامایی چه قدر است ؟ | فیزیکی-روانی | 4 |
|  |  |  |  |  | 21.امنیت شغلی درحرفه ی مامایی به لحاظ بازارکار چه قدر است ؟ |
|  |  |  |  |  | 22.کار در حرفه مامایی چقدر استرس زا است؟ |
|  |  |  |  |  | 23.چقدر مایلید در آینده در حرفه مامایی بمانید؟ |
|  |  |  |  |  | 24.وضعیت جایگاه اجتماعی حرفه مامایی در جامعه را چگونه ارزیابی می کنید؟(شاخصی عینی از وضعیت زندگی، درآمد و سطح تحصیلات فرد است که تعیین کننده طبقه اجتماعی یک فرد در جامعه است؟ | اجتماعی | 5 |
|  |  |  |  |  | 25.مقبولیت اجتماعی حرفه مامایی در جامعه را چگونه ارزیابی می کنید؟ (ارزیابی ذهنی که فرد از جایگاه خود در سلسه مراتب اجتماعی نسب به سایرین احساس می کند؟ |

**دیرکوند مقدم و همکاران، 1398**
