# Supplementary material for: Development and psychometric properties of Iranian midwives job satisfaction instrument (MJSI): A sequential exploratory study
Source: PLoS One. 2022 Jan 25;17(1):e0262665. doi: 10.1371/journal.pone.0262665 (PMC8789179; doi:10.1371/journal.pone.0262665)
Supplement: S6 File — (DOC) [file pone.0262665.s006.doc]

**Manual for scoring the Iranian midwives job satisfaction instrument(MJSI)**

|  | **Number of items** | **Minimum possible raw score** | **Maximum possible raw score** |
| --- | --- | --- | --- |
| **Communications dimension** | **7 (item 1-7)** | **0** | **28** |
| **Professional dimension** | **10 (item 8-17)** | **0** | **40** |
| **Responsibility dimension** | **2 (item 18-19)** | **0** | **8** |
| **Physical-Mental dimensions** | **4(item 20-23)** | **0** | **16** |
| **Social dimension** | **2(item 24-25)** | **0** | **8** |

**To calculate each subscale or total score for the MJSI, first we added raw scores and linearly transferred it to a score from 0 to 100 using the following formula.**

**Score=
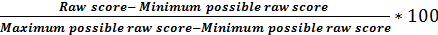
**

**©** [Direkvand-Moghadam](https://www.ncbi.nlm.nih.gov/pubmed/?term=Direkvand-Moghadam A%5BAuthor%5D&cauthor=true&cauthor_uid=22717414) A. et al., 2019
