# Supplementary material for: Development and psychometric properties of Iranian midwives job satisfaction instrument (MJSI): A sequential exploratory study
Source: PLoS One. 2022 Jan 25;17(1):e0262665. doi: 10.1371/journal.pone.0262665 (PMC8789179; doi:10.1371/journal.pone.0262665)
Supplement: Attachment — Submitted filename: commented.docx [file pone.0262665.s007.docx]

**Major revision comments**

1. Abstract

In the methodology part there are two types of study (design qualitative and crass sectional study design). What type’s method used to select the participants during crass -sectional study design?

**Result:** there is no any indication whether those four factors (communication, professional features, responsibility, and physical, mental, and social aspects) are they significantly affect the job satisfaction or variables used to assess the job satisfaction? Please rephrase the sentence

Validity and reliability no need of used in the result part

2. Methodology: developments of job satisfaction, the word educayion is incorrect, like this there are a lot of words which are incorrect so need of deep correction.
3. **Phase 2: Psychometric evaluation**:

You have been used to both qualitative and quantitative methods were used to determine the face validity. There different types of qualitative and quantitative methods, what types of study method did you apply for this study? No clear method

4. The calcification of very important (5), important (4), relatively important (3), slightly important (2), and unimportant was considered. The word calcification or classification I think you used for classification please correct it.where did you get such classification .are they prepared by yourself? If you have any source please put the reference
5. There is no any operational definition .why didn’t you put?
6. Discussion

Line 17 and 22 the reference style not correct just you are used the Vancouver reference style, please check the style.

7 All tables are	illegible. Use line to discriminate each variable
