# Supplementary material for: Development and psychometric properties of Iranian midwives job satisfaction instrument (MJSI): A sequential exploratory study
Source: PLoS One. 2022 Jan 25;17(1):e0262665. doi: 10.1371/journal.pone.0262665 (PMC8789179; doi:10.1371/journal.pone.0262665)
Supplement: Attachment — Submitted filename: Response to Reviewers.docx [file pone.0262665.s009.docx]

6. Review Comments to the Author

Please use the space provided to explain your answers to the questions above. You may also include additional comments for the author, including concerns about dual publication, research ethics, or publication ethics. (Please upload your review as an attachment if it exceeds 20,000 characters)

Reviewer #2: Thanks for invitation and giving extra time.
in th e Abstract some spelling error...like Crass sectional to be cross sectional. It was corrected. Abstract section. Line 31 and 32.

Reviewer #4: I want to thanks for getting this chance to review your document. I have got it well written and interesting topic. However, it is better to consider the following issues.
• It is better to take the last sentence of the first paragraph to the end of background section. It was corrected. background section. Line 56.

• In phase I: why being a faculty member or a midwife with at least 5 years of experience included? Why 5 year is selected? Why not more or less? In the explanation it should be said that According to the work experience of researchers and also consulting with experts in this field and according to the specific issue of job satisfaction in order to obtain the best results from the qualitative section of key informants, the inclusion criterion of at least 5 years of work experience was considered.

• Ethical statement is described in two place, in method section and in declaration section. Please avoid one of it based on journal format. It was corrected. Acknowledgments section. line 349.

• The discussion section is not in line with objective and findings. The second paragraph of discussion is typically background. The study used in the fourth paragraph of the discussion has no the same objective with your study. Therefore, it is impossible comparing these two study. the discussion have no justification for the similarity and difference occurred with other studies.
⎫ Overall, the discussion is somewhat seems poor, try two modify it.

Dear Reviewer

Hi, we tried very hard to write a worthy discussion, even though there was no tool for job satisfaction in the midwifery group and only one manuscript in the medical sciences in the field of tools. We hope it is satisfactory. It was corrected. Discussion section. Line 231-319.

• The sum of participants in table 1is not 121. Some are 120 some are 121. Why this difference occurred? . It was corrected. Table 1. line 405.

Reviewer #5: Thank you so much o give me an opprtunity ti re-review the study. the authors have perfomed the corrections carefully.
thre are only two comments: 1) I think it is better to report alpha cronbach for each domain not total. It was corrected. result section. line 214-216.
2) it ould be good if the authors put the persian of questionre as supplemntry file. Applied to the manuscript. It was placed in the supplementary part.
Good luck

7. PLOS authors have the option to publish the peer review history of their article ([what does this mean?](https://journals.plos.org/plosone/s/editorial-and-peer-review-process#loc-peer-review-history)). If published, this will include your full peer review and any attached files.


If you choose “no”, your identity will remain anonymous but your review may still be made public.


**Do you want your identity to be public for this peer review?** For information about this choice, including consent withdrawal, please see our [Privacy Policy](https://www.plos.org/privacy-policy).

Reviewer #2: **Yes:** Tiruset Gelaw(BSc, MSc in clinical midwifery)

Reviewer #4: No

Reviewer #5: **Yes:** Dr Leila Amiri-Farahani
